# The Emergence and Molecular Characteristics of New Delhi Metallo *β*-Lactamase-Producing *Escherichia coli* From Ducks in Guangdong, China

**DOI:** 10.3389/fmicb.2021.677633

**Published:** 2021-07-05

**Authors:** Min-Ge Wang, Yang Yu, Dong Wang, Run-Shi Yang, Ling Jia, Da-Tong Cai, Si-Lin Zheng, Liang-Xing Fang, Jian Sun, Ya-Hong Liu, Xiao-Ping Liao

**Affiliations:** ^1^National Risk Assessment Laboratory for Antimicrobial Resistance of Animal Original Bacteria, South China Agricultural University, Guangzhou, China; ^2^Laboratory of Veterinary Pharmacology, College of Veterinary Medicine, South China Agricultural University, Guangzhou, China; ^3^Guangdong Provincial Key Laboratory of Microbial Safety and Health, Guangdong Institute of Microbiology, Guangdong Academy of Science, Guangzhou, China; ^4^Guangdong Laboratory for Lingnan Modern Agriculture, Guangzhou, China

**Keywords:** transmission, MCR-1, *escherichia coli*, duck, *bla*_NDM_

## Abstract

This study aimed to determine the prevalence and transmission characteristics of New Delhi metallo β-lactamase (NDM)-producing *Escherichia coli* from ducks in Guangdong, China. In this study, a total of 28 NDM-producing *E. coli* isolates were recovered from 88 unduplicated diseased duck samples (31.8%) from veterinary clinics in Guangzhou, Foshan, Qingyuan, and Huizhou. Two variants, *bla*_NDM−1_ and *bla*_NDM−5_, were detected and the latter was present in 89.6% of the isolates (25/28). Multilocus sequence typing (MLST) analysis indicated that these *E. coli* isolates possessed six distinct STs, and ST156 was the most prevalent followed by ST648, ST746, ST354, ST10, and ST162. In addition, phylogenomic analysis found that two of the isolates that were recovered from a single sample possessed different genomes, and the *bla*_NDM_-carrying IncX3 plasmids may be horizontal transfer between *E. coli* isolates in the intestinal tracts of ducks. Whole-genome sequencing (WGS) analysis further revealed that *bla*_NDM_ co-existed with other 25 types of antimicrobial resistance genes (ARGs), of which 16 ARGs were highly prevalent with detection rates >50%, and a high incidence of coproducing *bla*_NDM_ and *mcr-1 E. coli* isolates (22/88, 25.0%) was detected in ducks. This study underscores the importance of surveillance for *bla*_NDM_-harboring microbes in ducks.

## Introduction

Carbapenems are critically important for the treatment of infections caused by multidrug-resistant Gram-negative bacteria. However, carbapenemase-producing *Enterobacteriaceae* have become a major global public health threat (Nordmann et al., 2011). In particular, the New Delhi metallo β-lactamase (NDM) was initially found in *Escherichia coli* and *Klebsiella pneumoniae* isolates in India in 2018 (Yong et al., 2009). Since then, *bla*_NDM_-positive isolates have been found globally (Wu et al., 2019). *bla*_NDM_ genes have been found in species belonging to 11 bacterial families, and the *Enterobacteriaceae* are the major hosts of *bla*_NDM_ (Wu et al., 2019; Zhai et al., 2020).

Carbapenem antibiotics have never been licensed for veterinary use in any country worldwide; however, there have been sporadic reports of *bla*_NDM_-positive isolates from a variety of animal hosts. *bla*_NDM_-positive *E. coli* isolates were frequently detected from swine in multiple geographic areas in China (Ho et al., 2019). Similar to the detected rates of *bla*_NDM_ gene in swine, *bla*_NDM_-positive *E. coli* isolates were highly prevalent in commercial broiler farms (Wang et al., 2017). In addition, several *bla*_NDM_-positive *E. coli* isolates from backyard animals shared closely related core single nucleotide polymorphisms (SNP) with human isolates (Li et al., 2019). A recent study has reported that *bla*_NDM_-positive *Enterobacteriaceae* were detected from migratory birds in China (Liao et al., 2019). Therefore, continued monitoring for *bla*_NDM_-positive Enterobacteriaceae in food-producing animals is urgently required.

Duck production has the potential to play a major role in the agricultural economy, and Asian countries alone contribute 84.2% of total duck meat produced in the world (Biswas et al., 2019). According to Food and Agriculture Organization (FAO) data (2019), duck meat production rose from 2.64 to 3.02 million tons from 2015 to 2019 in Asia, while in China, duck meat production rose from 2.19 to 2.50 million tons from 2015 to 2019 in China (FAO, 2019). The data show that China is the largest producer and consumer of cultivated duck in the world (Chen et al., 2020). *bla*_NDM_-positive *E. coli* isolates were highly prevalent along the Chinese poultry production chain, including commercial broiler farms, slaughterhouses, and supermarkets (Wang et al., 2017). Therefore, the prevalence of *bla*_NDM_-positive *E. coli* isolates from duck should be addressed through continued monitoring.

Although we previously reported that the rate of *bla*_NDM_-positive *E. coli* isolates at three duck farms in Guangdong was significantly higher than four other provinces in China, the sample collected was limited in western Guangdong province (Wang et al., 2021). Thus, in this study, we furthermore examined the epidemiology and molecular characterization of *bla*_NDM_-positive *E. coli* isolates recovered from ducks in representative areas for breeding ducks of Guangdong, China.

## Materials and Methods

### Ethics Statement

The Institutional Review Board of South China Agricultural University (SCAU-IRB) approved the protocols. All animals were sampled under authorization from the Animal Research Committees of South China Agricultural University (SCAU-IACUC).

### Sampling Information

**Figure 1:**
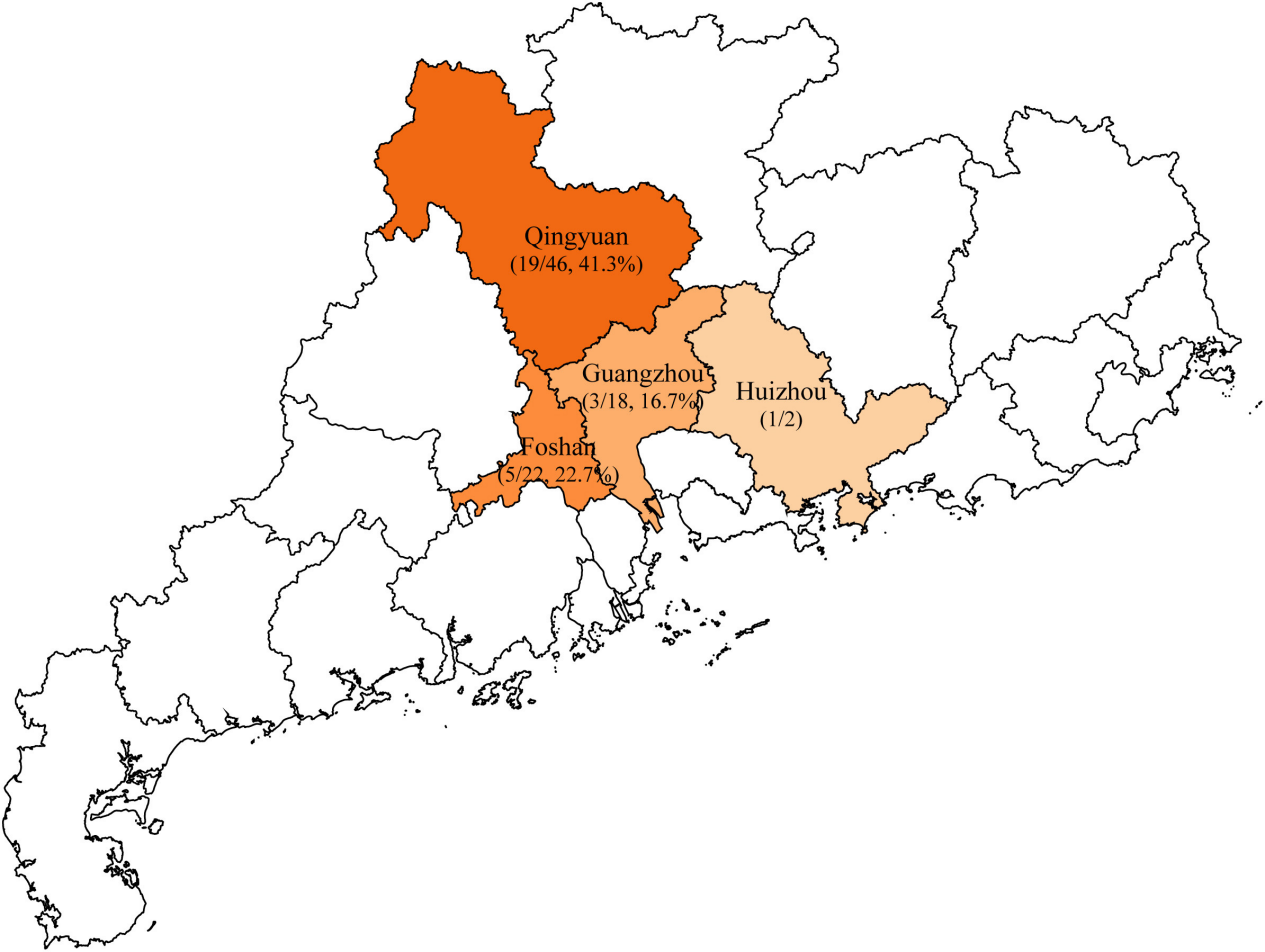
Sample collection areas in Guangdong, China. Municipalities included in the study are shaded in orange and the rate of *bla*_NDM_-positive *Escherichia coli* isolates are indicated by the depth of color.

A total of 88 unduplicated samples, including 42 liver samples and 46 caecum samples, were collected from 88 diseased ducks, these diseased ducks were sent to the veterinary clinical diagnostics laboratory in Foshan University from duck farms in Guangzhou, Huizhou, Foshan, and Qingyuan of Guangdong province (Figure 1). Briefly, all sample was added to 1 ml of LB Broth and incubated for 16–18 h in 37°C, followed by inoculating on MacConkey plates containing 2 mg/L meropenem for 12 h. Multiple red clones were selected for identification using MALDI-TOF MS Axima™ (Shimadzu-Biotech Corp., Kyoto, Japan) and 16S rRNA sequencing. For carbapenem-resistant *E. coli* isolates, five major carbapenem resistance genes, namely, *bla*_KPC_, *bla*_NDM_, *bla*_IMP_, *bla*_OXA−48_, and *bla*_VIM_, were detected by PCR using previously described primers (Poirel et al., 2011). Samples were collected after obtaining consent from farms and veterinarians.

### Antimicrobial Susceptibility Testing

Antibiotic susceptibility testing was performed by the agar dilution method and interpreted according to the Clinical and Laboratory Standards Institute guidelines (CLSI M100-S28) for the following antimicrobials: gentamicin, amikacin, meropenem, imipenem, aztreonam, cefotaxime, ceftazidime, cefoxitin, florfenicol, ciprofloxacin, fosfomycin, trimethoprim-sulfamethoxazole, and tetracycline (CLSI, 2018). Susceptibility to colistin and tigecycline were assessed by broth microdilution as recommended by the European Committee on Antimicrobial Susceptibility Testing (EUCAST Version 6.0) (EUCAST, 2016). *E. coli* ATCC 25922 was used as the quality control strain.

### Conjugation Assay and Southern Blotting

To investigate the transferability of the resistance genes, a conjugation assay was performed for all *bla*_NDM_-positive *E. coli* isolates with the streptomycin-resistant *E. coli* C600 as the recipient strain. Donor strains and *E. coli* C600 were mixed and applied to a 0.22 μm filter in LB agar plates for 16–18 h. The mixture culture was then diluted and spread on selective MacConkey agar plates containing both 1 mg/L of meropenem and 2,000 mg/L of streptomycin to recover transconjugants. Transconjugants were confirmed by PCR and Pulsed Field Gel Electrophoresis (PFGE) patterns. S1-PFGE and Southern blotting were performed to obtain plasmid size, and the *Salmonella enterica* serotype, Braenderup H9812, was used as the standard size marker.

### Whole-Genome Sequencing (WGS)

Total DNA was extracted from *bla*_NDM_-producing *E. coli* isolates using a Genomic DNA Purification Kit (TIANGEN, Beijing, China) as per the instructions of the manufacturer. WGS was performed with the Illumina Hiseq 2500 System (Novogene Guangzhou, China) using the paired-end 2 × 150-bp sequencing protocol. The draft genome was *de novo* assembled using the SPAdes version 3.9.0 (Bankevich et al., 2012). All genome assemblies of 28 *E. coli* isolates were deposited in GenBank and are registered with BioProject number PRJNA669620. Then, the sequence types, replicon types, and antibiotic resistance genes of all the isolates were identified by the Center for Genomic Epidemiology (http://www.genomicepidemiology.org/). The sequence comparison of *bla*_NDM_-carrying plasmids was performed using Mauve and Brig (Darling et al., 2004; Alikhan et al., 2011). *In silico* phylotyping of *E. coli* was carried out using the ClermonTyping method (http://clermontyping.iame-research.center/). Phylogenetic trees for the *bla*_NDM_-producing *E. coli* isolates were structured using CSI Phylogeny (v1.4), and *E. coli* (18FS1-1) was used as the reference genome (Kaas et al., 2014). The corresponding characteristics of each isolate were visualized using the online tool iTOL (https://itol.embl.de/). The population structure of each phylogenetic tree was defined using rhierbaps (Tonkin-Hill et al., 2018). The genome assemblies were analyzed using a gene-by-gene approach and the allelic distance from the core genome multilocus sequence typing (MLST) (cgMLST) was visualized in a minimum-spanning tree using BacWGSTdb 2.0 (Feng et al., 2021).

## Result

### Bacterial Isolation and Detection of Carbapenemase Genes

In this study, a total of 28 (31.8%) *bla*_NDM_-producing *E. coli* isolates were recovered from 88 collected samples in the veterinary clinical diagnostic laboratory in Foshan University from duck farms in Guangdong province. The isolation rates of *bla*_NDM_-positive *E. coli* from different districts were 41.3% for Qingyuan (19/46), 22.7% for Foshan (5/22), and 16.7% for Guangzhou (3/18) (Figure 1). Within these groups, we identified only two *bla*_NDM_ variants: *bla*_NDM−5_ (25/28, 89.3%) and *bla*_NDM−1_ (3/28, 10.7%) (Table 1).

**Table 1:** Bacterial information and antimicrobial resistance profiles.

**Isolates**	**Samples**	**Collection data**	**Sources**	**Variants**	***bla*_**NDM−**_carrying-**	**Resistance phenotype**
					**Plasmid**	
18FS1-1	18FS1	20160320	Caecum	*bla*_NDM−5_	IncX3	GEN, IMP, ERT, ATM, CTX, CAZ, FOX, CIP, FFC, TET, S/T
18FS1-2	18FS1	20160320	Caecum	*bla*_NDM−5_	IncX3	GEN, AMK, IMP, ERT, CTX, CAZ, FOX, CIP, FFC, TET, S/T, CS
18FS2-1	18FS2	20160320	Liver	*bla*_NDM−5_	IncX3	IMP, ERT, CTX, CAZ, FOX, CIP, FFC, TET, S/T, FOS
18FS3-1	18FS3	20160320	Liver	*bla*_NDM−5_	IncX3	GEN, IMP, ERT, CTX, CAZ, FOX, CIP, FFC, TET, FOS
18FS4-1	18FS4	20160320	Caecum	*bla*_NDM−5_	IncX3	GEN, IMP, ERT, CTX, CAZ, FOX, CIP, FFC, TET
18FS4-2	18FS4	20160320	Caecum	*bla*_NDM−5_	IncX3	IMP, ERT, CTX, CAZ, FOX, CIP, FFC, TET
18FS5-2	18FS5	20160320	Liver	*bla*_NDM−5_	IncX3	IMP, ERT, CTX, CAZ, FOX, CIP, FFC, TET
18FS7-1	18FS7	20160320	Caecum	*bla*_NDM−5_	IncX3	GEN, IMP, ERT, CTX, CAZ, FOX, CIP, FFC, TET, S/T, FOS
18FS7-2	18FS7	20160320	Caecum	*bla*_NDM−5_	IncX3	GEN, IMP, ERT, CTX, CAZ, FOX, CIP, FFC, TET, S/T
18FS7-3	18FS7	20160320	Caecum	*bla*_NDM−5_	IncX3	GEN, IMP, ERT, CTX, CAZ, FOX, CIP, FFC, TET, S/T
18FS15-1	18FS15	20160320	Caecum	*bla*_NDM−5_	IncX3	GEN, IMP, ERT, CTX, CAZ, FOX, CIP, FFC, TET, S/T
18FS16-2	18FS16	20160320	Liver	*bla*_NDM−5_	IncX3	GEN, IMP, ERT, CTX, CAZ, FOX, CIP, FFC, TET, FOS
18FS16-3	18FS16	20160320	Liver	*bla*_NDM−5_	IncX3	GEN, AMK, IMP, ERT, ATM, CTX, CAZ, FOX, CIP, FFC, TET, S/T
18FS17-3	18FS17	20160320	Caecum	*bla*_NDM−5_	IncX3	GEN, IMP, ERT, ATM, CTX, CAZ, FOX, CIP, FFC, TET, S/T
18FS18-1	18FS18	20160320	Caecum	*bla*_NDM−5_	IncX3	GEN, IMP, ERT, ATM, CTX, CAZ, FOX, CIP, FFC, TET, S/T, CS
18FS18-2	18FS18	20160320	Caecum	*bla*_NDM−5_	IncX3	GEN, IMP, ERT, CTX, CAZ, FOX, CIP, FFC, TET
18FS23-1	18FS23	20160320	Caecum	*bla*_NDM−5_	IncX3	GEN, AMK, IMP, ERT, CTX, CAZ, FOX, CIP, FFC, TET, S/T
18FS24-1	18FS24	20160320	Liver	*bla*_NDM−5_	IncX3	GEN, IMP, ERT, CTX, CAZ, FOX, CIP, FFC, TET, S/T, FOS
20FS11-1	20FS11	20160402	Caecum	*bla*_NDM−5_	IncX3	GEN, AMK, IMP, ERT, CTX, CAZ, FOX, CIP, FFC, TET, S/T
20FS11-2	20FS11	20160402	Caecum	*bla*_NDM−1_	Untypable	GEN, AMK, IMP, ERT, ATM, CTX, CAZ, FOX, CIP, FFC, TET, S/T, CS, FOS
20FS12-2	20FS12	20160402	Liver	*bla*_NDM−1_	Untypable	IMP, ERT, CTX, CAZ, FOX, CIP, FFC, TET, S/T, CS, FOS
20FS14	20FS14	20160402	Liver	*bla*_NDM−1_	Untypable	GEN, AMK, IMP, ERT, ATM, CTX, CAZ, FOX, CIP, FFC, TET, S/T, CS
20FS19	20FS19	20160402	Caecum	*bla*_NDM−5_	IncX3	GEN, IMP, ERT, ATM, CTX, CAZ, FOX, CIP, FFC, TET, S/T
20FS22	20FS22	20160402	Liver	*bla*_NDM−5_	IncX3	IMP, ERT, CTX, CAZ, FOX, CIP, FFC, TET, S/T
21FS11-2	21FS11	20160409	Caecum	*bla*_NDM−5_	IncX3	GEN, IMP, ERT, CTX, CAZ, FOX, CIP, FFC, TET, S/T
22FS12-2	22FS12	20160416	Liver	*bla*_NDM−5_	IncX3	GEN, AMK, IMP, ERT, CTX, CAZ, FOX, CIP, FFC, TET, S/T, CS
22FS18	22FS18	20160416	Liver	*bla*_NDM−5_	IncX3	GEN, AMK, IMP, ERT, CTX, CAZ, FOX, CIP, FFC, TET, S/T, CS, FOS
22FS24	22FS24	20160416	Liver	*bla*_NDM−5_	IncX3	GEN, AMK, IMP, ERT, CTX, CAZ, FOX, CIP, FFC, TET, S/T, CS, FOS

*Samples were collected from the caecum and liver of ducks. MEM, meropenem; CTX, cefotaxime; CAZ, ceftazidime; FOX, cefoxitin; FFC, florfenicol; CIP, ciprofloxacin; TET, tetracycline, S/T, trimethoprim/sulfamethoxazole; IMP, imipenem; ERT, ertapenem; GEN, gentamicin; FOS, fosfomycin; CS, colistin; AMK, amikacin; ATM, aztreonam; TIG, tigecycline*.

### Antibiotic Susceptibility Testing

All *bla*_NDM_-positive *E. coli* isolates showed reduced susceptibility to meropenem with MICs of 4– >64 mg/L (Supplementary Table 1) and were concurrently resistant to imipenem, ertapenem, cefotaxime, ceftazidime, cefoxitin, florfenicol, ciprofloxacin, and tetracycline (Table 1). Moreover, these isolates exhibited high rates of resistance to trimethoprim/sulfamethoxazole (22/28, 78.6%), gentamicin (23/28, 82.1%), and fosfomycin (12/28, 42.9%) but lower rates for amikacin (9/28, 32.1%), colistin (8/28, 28.6%), and aztreonam (7/28, 25%).

### Phylogenetic Analysis of NDM-Positive *E. coli* Isolates

**Figure 2:**
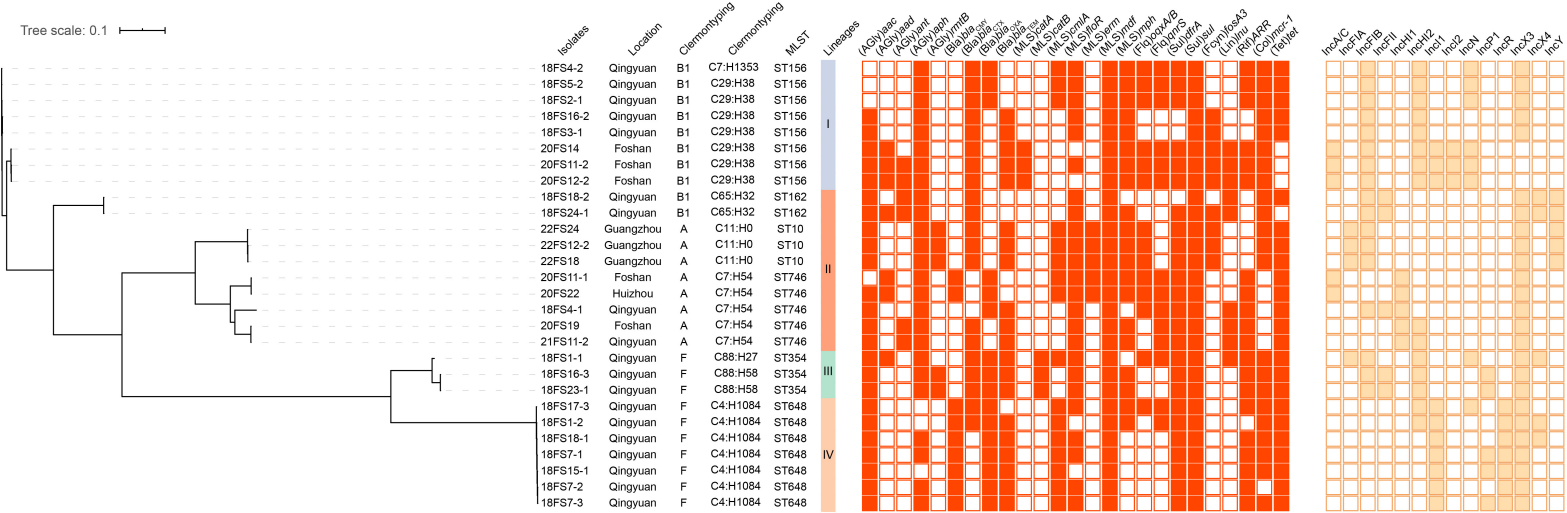
Analysis of *bla*_NDM_-positive *E. coli* isolates from ducks in Guangdong, China. Relationships among 28 *bla*_NDM_-positive *E. coli* isolates are indicated using a maximum likelihood tree. Red-filled squares indicate possession of the indicated antimicrobial resistance genes (ARGs) and brown-filled squares indicated plasmid Inc type.

Whole-genome sequencing data were generated for the 28 *bla*_NDM_-positive *E. coli* isolates. The results of WGS demonstrated that these isolates were divided into six distinct STs: ST156 (8/28, 28.6%), ST648 (7/28, 25.0%), ST746 (5/28, 17.9%), ST354 (3/28, 10.7%), ST10 (3/28, 10.7%), and ST162 (2/28, 7.1%) (Figure 2). Clonotyping revealed seven *fumC* and *fimH* (CH) types and exhibited further divergence between clones. The most prevalent clonotypes were C29:H38 (*n* = 7), C4:H1084 (*n* = 7), and C7:H54 (*n* = 5), which belong to ST156, ST648, and ST746, respectively. The remaining clonotypes were C11:H0 (*n* = 3), C65:H32 (*n* = 2), C88:H58 (*n* = 2), C7:H1353 (*n* = 1), and C88:H27 (*n* = 1) (Figure 2). The *E. coli* isolates from the present study were classified using Clermont Typing and the majority belonged to groups B1 (10/28, 35.7%) and F (10/28, 35.7%) from Foshan and Qingyuan, respectively. A phylogenetic tree was established using the 28 *bla*_NDM_-positive *E. coli* isolates. All isolates were classified into four clades lineage, and the major Lineage IV included seven (25%) isolates belonging to ST648 and exhibited high levels of the identity of pairwise single nucleotide polymorphisms (SNP) ≤ 21 (Figure 2). Notably, in five cases, two isolates were possessing a collection of different genomic characteristics that were recovered from the same samples (18FS1, 18FS4, 18FS16, 18FS18, and 20FS11) (Table 1). For instance, both ST354 (18FS1-1) and ST648 (18FS1-2) were recovered from sample 18FS1 and shared 22411 SNPs. This scenario is worrying and indicates the development of diversity in the population of *bla*_NDM_-positive *E. coli* isolates from ducks. To further assess the relationship between the 28 isolates, the genome assemblies were analyzed using a gene-by-gene approach, and the allelic distance from the core genome MLST (cgMLST) was visualized in a minimum-spanning tree using BacWGSTdb 2.0. Based on ST, geographic location, and position in the network, the resulting network shows that the isolates were grouped in accordance with their ST and place of isolation. The MLST type was clustered together, except ST746; 18FS4-1 were more closed to the ST156 cluster (Supplementary Figure 1).

### Antibiotic Resistance Genes

We additionally identified the presence of 25 types of antimicrobial resistance genes (ARGs) that conferred resistance to nine classes of antibiotics including aminoglycosides, β-lactams, MLS (macrolides, lincosamides, and type B streptogramin), fluoroquinolones, sulfonamide, fosfomycin, rifampicin, colistin, and tetracyclines. Among these, 16 ARGs were highly prevalent with detection rates >50%, including *aac, aph, bla*_CTX_, *bla*_OXA_, *bla*_TEM_, *cmlA, floR, mdf*, *mph, oqxA/B, qnrS, dfrA, sul, ARR, mcr-1*, and *tet* (Figure 2).

### Plasmid Analysis

Conjugation experiments were performed using the *bla*_NDM_-positive *E. coli* isolates collected from ducks, and all the *bla*_NDM_-carrying plasmids were successfully transferred to the recipient strain *E. coli* C600^str^. S1-PFGE and hybridization analyses confirmed that *bla*_NDM_ genes from 25 isolates were located on ~ 50 kb plasmids, and the others genes were located on ~ 140 kb (*n* = 2) and ~ 200 kb (*n* = 1) plasmids (Supplementary Figure 2). As shown in Supplementary Figure 3, the ~ 50 kb plasmids carrying *bla*_NDM_ were the IncX3 incompatibility group and were similar to *bla*_NDM−5_-carrying IncX3 plasmid from an ST25 *K. pneumoniae* isolated from human peritoneal fluid in China (Acc. No. KU761328). In addition, we found that IncX3 plasmids were carried by two different *E. coli* isolates that were recovered from the same sample in four out of five cases (Table 1). This provides evidence for the horizontal transfer of *bla*_NDM_-carrying IncX3 plasmids in the intestines of ducks. WGS analysis demonstrated that 14 different Inc types were present on plasmids in the 28 *bla*_NDM_-positive *E. coli* isolates (Figure 2). Except for the IncX3 plasmid, IncFIB (17/28, 60.7%), IncHI2 (15/28, 53.6%), and IncI1 (10/28, 35.7%) plasmids were highly prevalent in these isolates (Figure 2).

## Discussion

In this study, a total of 28 (28/88, 31.8%) *bla*_NDM_-producing *E. coli* isolates were recovered from ducks; this result is similar to our previous report that indicated ahigh prevalence of *bla*_NDM_ -positive *E. coli* isolates at duck farms in western Guangdong province. Although there was an overwhelming dominance of *bla*_NDM−1_ and *bla*_NDM−5_ in the clinical and livestock isolates (Shen et al., 2018; Zhai et al., 2020), *bla*_NDM−5_ was more prevalent than *bla*_NDM−1_ among the tested duck farms. This finding is similar to the previous report of the high prevalence of *bla*_NDM−5_ in the chicken production chains (Wang et al., 2017).

The 28 *bla*_NDM_-positive *E. coli* isolates belonged to six distinct STs (ST156, ST648, ST746, ST354, ST10, and ST162) discussed in the current study, of which, ST156, ST648, and ST746 were the most prevalent. ST156 and ST648 have been associated with the dissemination of *bla*_NDM−5_ and *mcr-1*-producing *E. coli* isolates (Yang et al., 2016). The *bla*_NDM_-positive ST746 and ST354 prevalent in ducks and poultry have become a primary reservoir for *bla*_NDM_-positive ST746 *E. coli* isolates in China (Wang et al., 2021). Phylogenomic analysis found that two of the isolates that were recovered from a single sample possessed different genomes, which indicates the development of diversity in the population of *bla*_NDM_-positive *E. coli* isolates from ducks.

Whole-genome sequencing analysis further revealed that *bla*_NDM_ coexisted with other 25 types of ARGs, of which 16 ARGs were highly prevalent with detection rates >50%. Of note, *mcr-1*, conferring resistance to the last-resort antibiotic colistin, was detected in 22 *bla*_NDM_-positive *E. coli* isolates. Some recent studies have reported that *bla*_NDM_ and *mcr-1* coproducing *E. coli* isolates were recovered from chicken (37/739, 5.0%), swine (16/105, 15.2%), and duck (11/92, 12.0%) farms (Kong et al., 2017; Wang et al., 2017, 2021). There was also a high incidence of the co-harboring of *mcr-1* and *bla*_NDM_ in chickens (21/78, 26.9%) (Liu et al., 2017). In this study, we also discovered a high prevalence of *bla*_NDM_ and *mcr-1* co-carrying *E. coli* isolates from diseased ducks (22/88, 25.0%) in Guangdong. In addition, the existence of *bla*_NDM−5_ is associated with multiple resistances, including aminoglycosides, sulfonamide, and fluoroquinolones. These can further promote the spread and persistence of carbapenem-resistant microbes in the poultry industry (Grönthal et al., 2018; Zhai et al., 2020).

In the current study, all *bla*_NDM−5_ genes identified were carried by IncX3 plasmids. IncX3 plasmids may serve as one of the major platforms on which *bla*_NDM_ genes are evolving with the generation of new NDM variants, such as *bla*_NDM−1/4/5/6/7/13/17/19/20/21_ (Wu et al., 2019; Zhai et al., 2020). However, a high prevalence of *bla*_NDM−5_-carrying IncX3 plasmid from bacteria of animal farms in China (Zhai et al., 2020). The *bla*_NDM_-carrying IncX3 plasmids have a narrow host range and have been mainly found in *Enterobacteriaceae* worldwide, which may be an association to highly conjugatable and stable and exert no fitness costs on their bacterial hosts (Johnson et al., 2012). In addition, this study found that IncX3 plasmids were carried with two different *E. coli* isolates recovered from a single sample in four out of five samples. This provides evidence for the horizontal transfer of *bla*_NDM_-carrying IncX3 plasmids between *E. coli* isolates in the intestine of ducks.

## Conclusions

In conclusion, we identified 28 *bla*_NDM_-positive *E. coli* isolates from diseased ducks in Guangdong, China. Notably, this is the first study to report the development of diversity in the population of *bla*_NDM_-positive *E. coli* isolates from ducks. WGS analysis further determined that *bla*_NDM_ coexisted with other ARGs, including *mcr-1*, and *bla*_NDM_-carrying IncX3 plasmids were most likely horizontally transferred between *E. coli* isolates in the duck intestinal tract. This study underscores the importance of surveillance for *bla*_NDM_-harboring microbes in ducks and indicates a high likelihood for the spread of carbapenem resistance from the poultry production chain to humans.

## Data Availability Statement

The datasets presented in this study can be found in online repositories. The names of the repository/repositories and accession number(s) can be found at: https://www.ncbi.nlm.nih.gov/genbank/, PRJNA669620.

## Author Contributions

M-GW wrote the first draft of the manuscript. X-PL, YY, and L-XF contributed to conception and design of the study. DW, R-SY, LJ, D-TC, and S-LZ performed the statistical analysis. JS and Y-HL wrote sections of the manuscript. All authors contributed to manuscript revision, read, and approved the submitted version.

## Conflict of Interest

The authors declare that the research was conducted in the absence of any commercial or financial relationships that could be construed as a potential conflict of interest.

## Supplementary Material

Supplementary Figure 1 Minimum spanning tree analysis of the cgMLST profiles of 28 isolates. Numbers on lines correspond to the number of target genes for which allelic differences were detected. Circle colors are used to differentiate the city of origin of the isolates.

Supplementary Figure 2 S1-PFGE and hybridization of the plasmids of *bla*_NDM_-positive *Escherichia coli* isolates. Lane M: XbaI-digested genomic DNA of reference *Salmonella enterica* serotype Braenderup strain H9812, and the bands on membrane represent the plasmids where *bla*_NDM_ genes are located on.

Supplementary Figure 3 The sequence comparison and map generation of *bla*_NDM_-carrying plasmids with reference IncX3 plasmid (Accession Number: KU761328) from an ST25 *Klebsiella pneumoniae* isolated from human peritoneal fluid in China.

Supplementary Table 1 Minimum inhibitory concentration of 28 *bla*_NDM_-positive E. coli isolates from duck in Guangdong China.
