# Supplementary figures and images for: The Emergence and Molecular Characteristics of New Delhi Metallo β-Lactamase-Producing Escherichia coli From Ducks in Guangdong, China
Source: Front Microbiol. 2021 Jul 5;12:677633. doi: 10.3389/fmicb.2021.677633 (PMC8287858; doi:10.3389/fmicb.2021.677633)

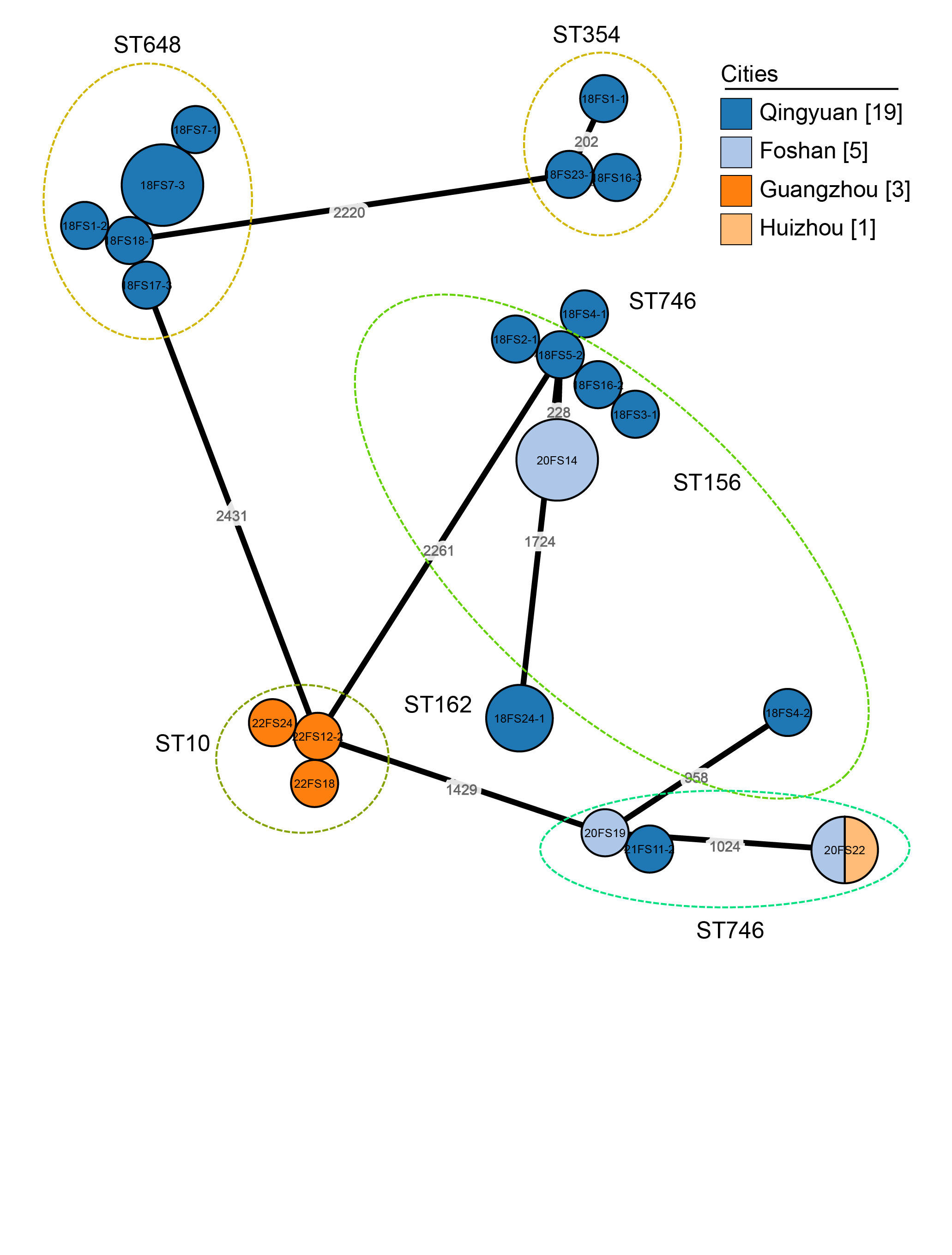

Supplement: Supplementary Figure 1 — Minimum spanning tree analysis of the cgMLST profiles of 28 isolates. Numbers on lines correspond to the number of target genes for which allelic differences were detected. Circle colors are used to differentiate the city of origin of the isolates. [file Image_1.JPEG]

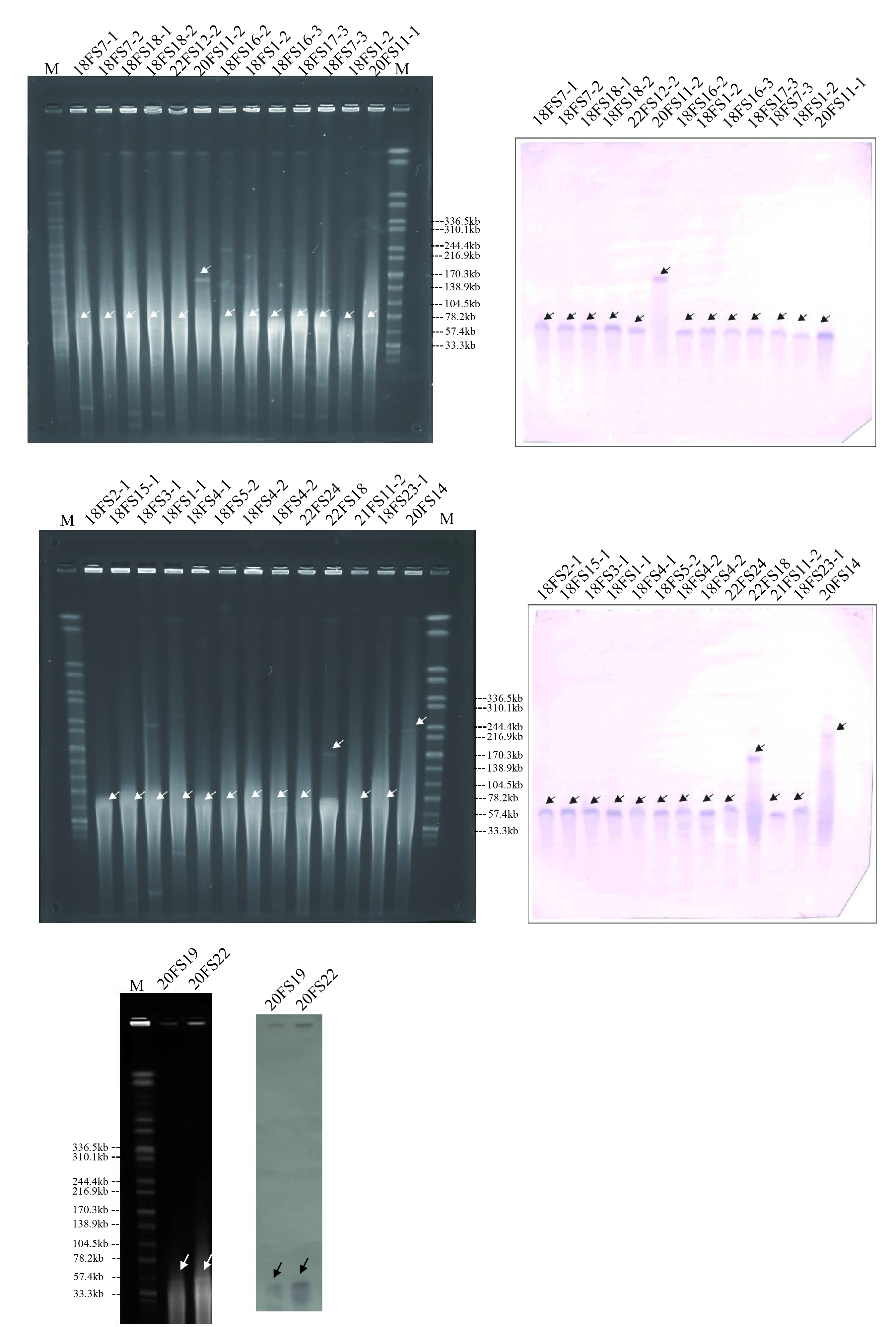

Supplement: Supplementary Figure 2 — S1-PFGE and hybridization of the plasmids of blaNDM-positive Escherichia coli isolates. Lane M: XbaI-digested genomic DNA of reference Salmonella enterica serotype Braenderup strain H9812, and the bands on membrane represent the plasmids where blaNDM genes are located on. [file Image_2.JPEG]

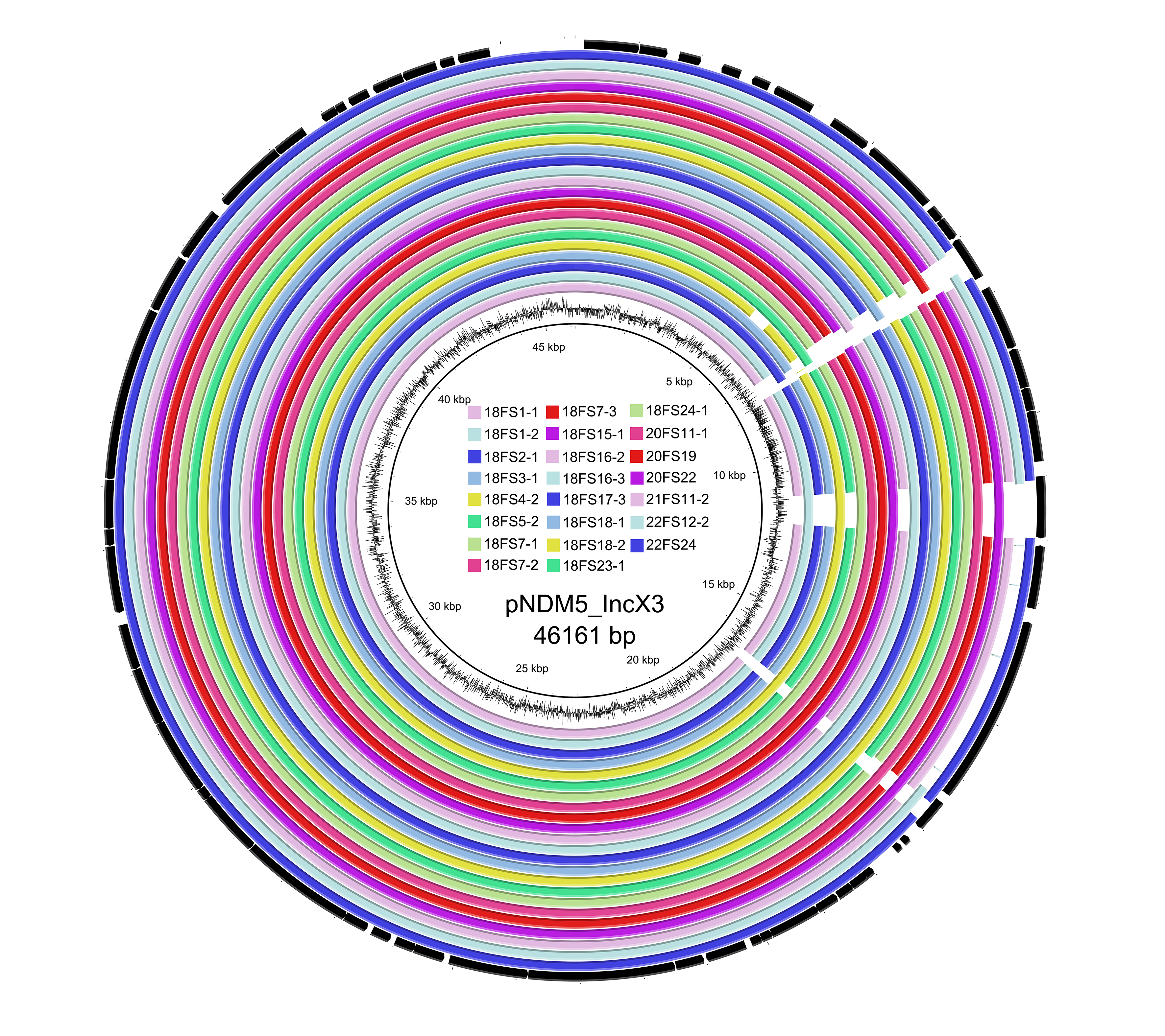

Supplement: Supplementary Figure 3 — The sequence comparison and map generation of blaNDM-carrying plasmids with reference IncX3 plasmid (Accession Number: KU761328) from an ST25 Klebsiella pneumoniae isolated from human peritoneal fluid in China. [file Image_3.JPEG]
