# Supplementary material for: The Emergence and Molecular Characteristics of New Delhi Metallo β-Lactamase-Producing Escherichia coli From Ducks in Guangdong, China
Source: Front Microbiol. 2021 Jul 5;12:677633. doi: 10.3389/fmicb.2021.677633 (PMC8287858; doi:10.3389/fmicb.2021.677633)
Supplement: Supplementary Table 1 — Minimum inhibitory concentration of 28 blaNDM-positive E. coli isolates from duck in Guangdong China. [file Table_1.DOCX]

| Isolate | GEN | AMK | IMP | ERT | MEM | ATM | CTX | CAZ | FOX | FOS | CIP | FFC | TET | S\T | CS |
| --- | --- | --- | --- | --- | --- | --- | --- | --- | --- | --- | --- | --- | --- | --- | --- |
| 18FS1-1 | 64 | 4 | 16 | 64 | 8 | 32 | >256 | >256 | >256 | 128 | 256 | 256 | 128 | >320 | 4 |
| 18FS1-2 | >256 | >256 | 16 | 64 | 8 | 1 | >256 | >256 | >256 | 128 | 256 | >256 | 64 | 160 | 8 |
| 18FS2-1 | 1 | 8 | 16 | >256 | 16 | 16 | >256 | >256 | >256 | 256 | >256 | >256 | 64 | >320 | 4 |
| 18FS3-1 | 16 | 4 | 16 | >256 | 16 | 2 | >256 | >256 | >256 | >256 | 256 | 128 | 64 | 1.25 | 4 |
| 18FS4-1 | 32 | 2 | 16 | >256 | 8 | 4 | >256 | >256 | >256 | 128 | >256 | 128 | 64 | 1.25 |  |
| 18FS4-2 | 8 | 4 | 32 | >256 | 16 | 8 | >256 | >256 | >256 | 128 | 256 | 128 | 64 | <1.25 | 4 |
| 18FS5-2 | 1 | 4 | 16 | >256 | 16 | 16 | >256 | >256 | >256 | 128 | >256 | >256 | 64 | 40 | 4 |
| 18FS7-1 | 32 | 8 | 32 | >256 | 32 | 16 | >256 | >256 | >256 | 256 | 256 | >256 | 256 | >320 | 0.5 |
| 18FS7-2 | 32 | 4 | 64 | >256 | 64 | 16 | >256 | >256 | >256 | 16 | 256 | >256 | 256 | >320 | 4 |
| 18FS7-3 | 64 | 4 | 64 | >256 | 32 | 16 | >256 | >256 | >256 | 16 | 128 | >256 | 256 | >320 | 0.25 |
| 18FS15-1 | 32 | 4 | 32 | >256 | 32 | 16 | >256 | >256 | >256 | 16 | 256 | 32 | 16 | >320 | 0.25 |
| 18FS16-2 | 16 | 8 | 16 | >256 | 16 | 2 | >256 | >256 | >256 | >256 | 256 | 256 | 64 | 1.25 | 4 |
| 18FS16-3 | >256 | >256 | 64 | >256 | 32 | 32 | >256 | >256 | >256 | 128 | >256 | 256 | 256 | >320 | 4 |
| 18FS17-3 | 32 | 4 | 64 | >256 | 32 | 32 | >256 | >256 | >256 | 32 | 256 | 128 | 128 | >320 | 0.25 |
| 18FS18-1 | 32 | 4 | 32 | >256 | 32 | 64 | >256 | >256 | >256 | 32 | 256 | >256 | 256 | 160 | 8 |
| 18FS18-2 | 256 | 4 | 16 | >256 | 16 | 8 | >256 | >256 | >256 | 16 | 32 | >256 | 128 | <1.25 | 4 |
| 18FS23-1 | >256 | >256 | 32 | >256 | 32 | 16 | >256 | >256 | >256 | 16 | >256 | >256 | 256 | >320 | 4 |
| 18FS24-1 | 64 | 4 | 16 | 64 | 8 | 8 | >256 | >256 | >256 | >256 | 32 | 256 | 128 | >320 | 4 |
| 20FS11-1 | >256 | >256 | 16 | 32 | 4 | 32 | >256 | >256 | >256 | 256 | 128 | >256 | 64 | >320 | 8 |
| 20FS12-2 | 1 | 8 | 16 | >256 | 16 | 16 | >256 | >256 | >256 | 256 | >256 | >256 | 64 | 320 | 8 |
| 20FS14 | >256 | >256 | 16 | >256 | 16 | 32 | >256 | >256 | >256 | >256 | 256 | >256 | 256 | >320 | 8 |
| 20FS19 | >256 | 4 | 16 | 64 | 16 | 128 | >256 | >256 | >256 | 64 | 128 | >256 | 256 | >320 | 0.25 |
| 20FS22 | 1 | 8 | 32 | >256 | 32 | 8 | >256 | >256 | >256 | 128 | >256 | >256 | 256 | >320 | 0.25 |
| 21FS11-2 | 64 | 4 | 32 | >256 | 32 | 16 | >256 | >256 | >256 | 128 | 256 | >256 | 256 | >320 | 0.25 |
| 22FS12-2 | >256 | >256 | 16 | 32 | 8 | 1 | >256 | >256 | >256 | 128 | 64 | >256 | 64 | >320 | 8 |
| 22FS18 | >256 | >256 | 16 | 32 | 16 | 2 | >256 | >256 | >256 | >256 | 64 | >256 | 64 | >320 | 8 |
| 22FS24 | >256 | >256 | 32 | 32 | 4 | 2 | >256 | >256 | >256 | >256 | 128 | >256 | 64 | >320 | 8 |

Table S1: Minimum inhibitory concentration of 28 blaNDM-positive E. coli isolates from duck in Guangdong China.
